# First person – Casandra Newkirk

**DOI:** 10.1242/bio.059583

**Published:** 2022-09-13

## Abstract

First Person is a series of interviews with the first authors of a selection of papers published in Biology Open, helping researchers promote themselves alongside their papers. Casandra Newkirk is first author on ‘ Reproducible propagation technique for the symbiotic cnidarian model system Cassiopea xamachana’, published in BiO. Casandra is a postdoctoral fellow in the lab of Tingting Xiang, PhD, at the University of North Carolina at Charlotte, investigating marine ecology with a focus on cnidarian symbiosis.


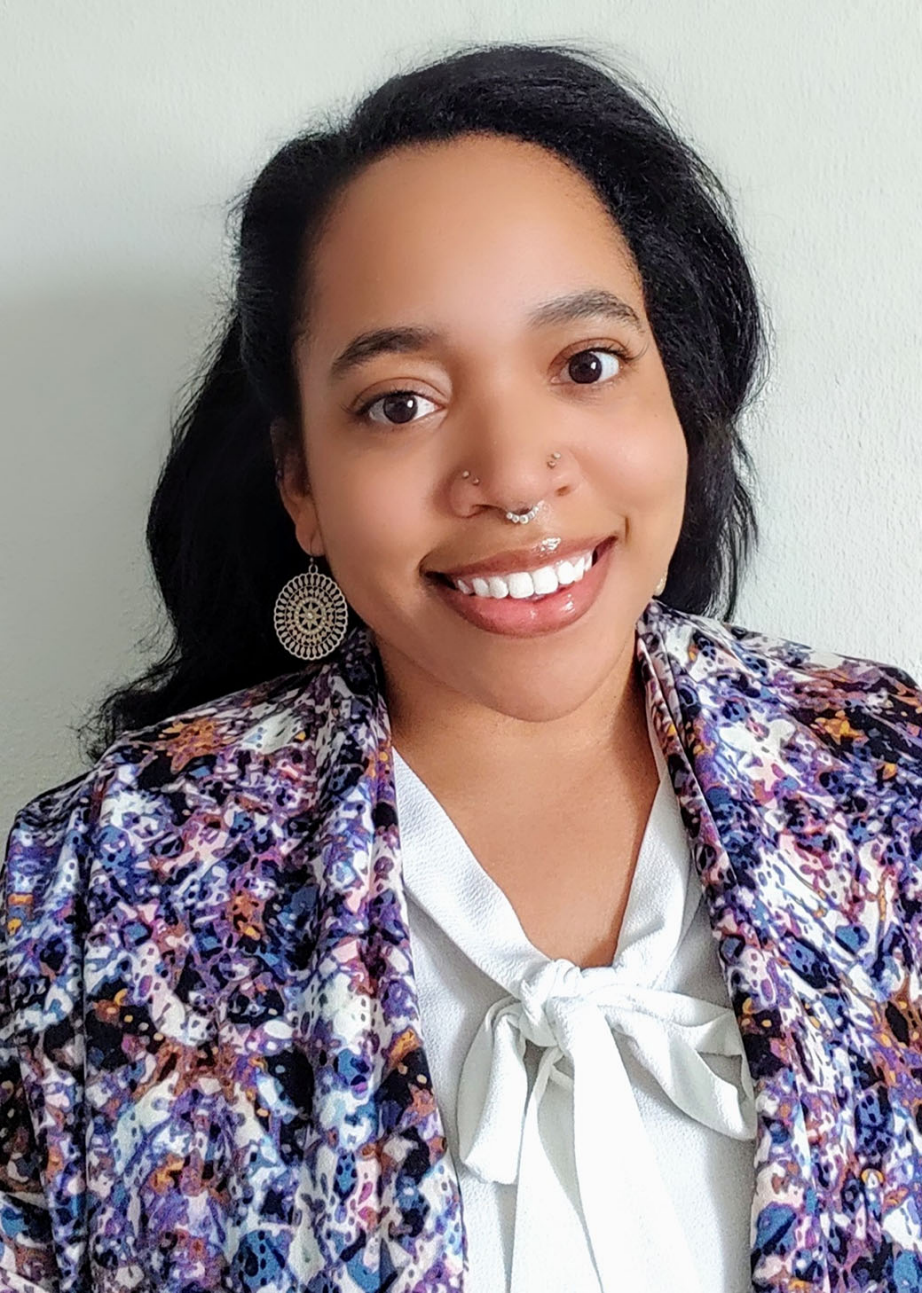


**Casandra Newkirk**

**Describe your scientific journey and your current research focus**

I got started in laboratory research during my sophomore year of college. I continued my journey throughout college and worked at two different marine laboratories as an intern during this time (The Whitney Laboratory for Marine Bioscience and The Marine Biological Laboratory). These experiences helped solidify my interest in marine science, and when I was accepted into graduate school at The University of Florida I knew that was a path I wanted to continue on. I have been studying cnidarian symbiosis since 2014, with my primary research organism being *Cassiopea xamachana* (a.k.a. the upside-down jellyfish). I am interested in understanding how the symbiosis between the dinoflagellate algae that lives within the tissues of the jellyfish is impacted by environmental stressors such as high temperatures.

**Who or what inspired you to become a scientist?**

I have wanted to be a scientist ever since I was young. Marine science became a passion after my first visit to an aquarium.

**How would you explain the main finding of your paper?**

This paper outlines a way to quickly grow the number of juvenile *Cassiopea* specimens using a cutting/dissection technique. This method of propagation will be useful to helping broaden the use of *Cassiopea* as a model organism for experimental laboratory use.

**What are the potential implications of this finding for your field of research?**

This new method can help propagate *Cassiopea* under any laboratory conditions; it can also provide another method for acquiring clonal lines of *Cassiopea* polyps.

**Figure:**
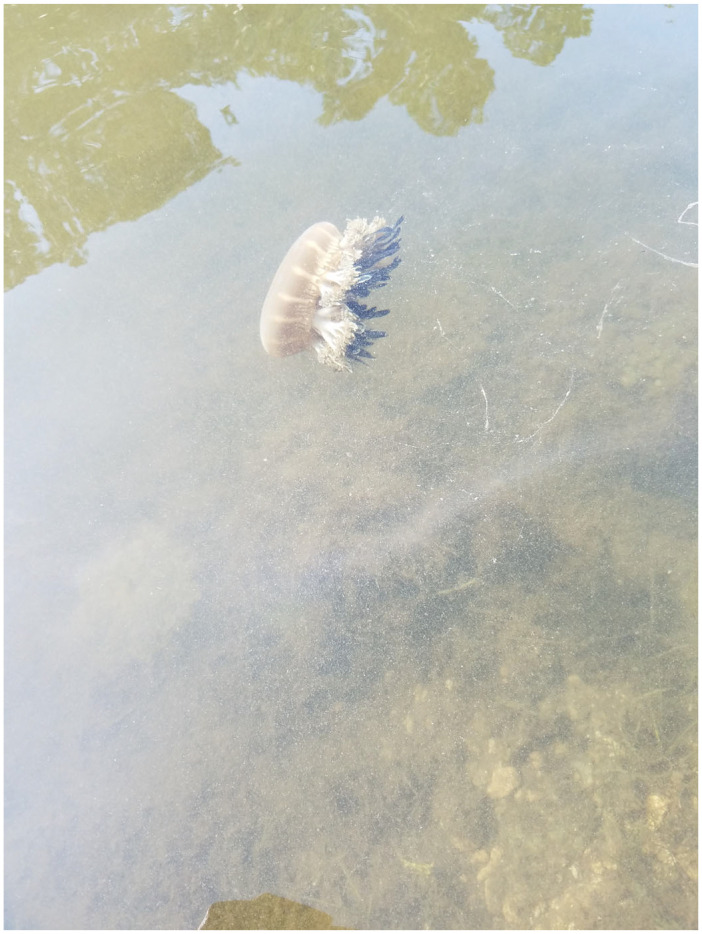
A Cassiopea adult swimming home.

**Which part of this research project was the most rewarding?**

The most rewarding part of this manuscript was working with the three undergraduate students that are now listed as co-authors. These students worked hard and got a first-hand account of how to follow a research project through from beginning to end. I am very proud of this team!

**What do you enjoy most about being an early-career researcher?**

I enjoy the ability to not only be able to carry out research, but also the opportunity to be able to mentor others. Good mentorship when I was a student is the top reason I got all of the opportunities I was awarded. I hope to be able to do that same for the students under my mentorship and my mentors did (and continue) to do for me.

**What piece of advice would you give to the next generation of researchers?**

Be persistent in your goals, but also maintain a fulfilling life outside of science. A good balance between the two will allow you to be a more well-rounded (and rested) scientist.

**What's next for you?**

I am not 100% sure what is next for me. I would love to continue in a position that allows me to do science communication and scientific outreach, as these are two of my main passions.

## References

[BIO059583C1] Newkirk, C., Vadlapudi, S., Sadula, M., Arbello, C. and Xiang, T. (2022). Reproducible propagation technique for the symbiotic cnidarian model system Cassiopea xamachana. *Biol. Open* 11, bio059413. 10.1242/bio.059413 36066114 PMC9493721

